# Smooth Muscle Specific Ablation of CXCL12 in Mice Downregulates CXCR7 Associated with Defective Coronary Arteries and Cardiac Hypertrophy

**DOI:** 10.3390/ijms22115908

**Published:** 2021-05-31

**Authors:** Santhosh Kumar Ghadge, Moritz Messner, Herbert Seiringer, Thomas Maurer, Simon Staggl, Tanja Zeller, Christian Müller, Daniela Börnigen, Wolfgang J. Weninger, Stefan H. Geyer, Sieghart Sopper, Anne Krogsdam, Gerhard Pölzl, Axel Bauer, Marc-Michael Zaruba

**Affiliations:** 1 Department of Internal Medicine III, Cardiology and Angiology, Medical University Innsbruck, 6020 Innsbruck, Austria; santhosh.ghadge@meduniwien.ac.at (S.K.G.); moritz.messner@i-med.ac.at (M.M.); herbert.seiringer@student.i-med.ac.at (H.S.); thomas.maurer@student.i-med.ac.at (T.M.); simon.staggl@i-med.ac.at (S.S.); gerhard.poelzl@i-med.ac.at (G.P.); axel.bauer@i-med.ac.at (A.B.); 2 Department of Medical Biochemistry, Max F. Perutz Laboratories (MFPL), Medical University of Vienna, 1090 Vienna, Austria; 3 Clinic for Cardiology, Medical University Center Hamburg-Eppendorf, University Heart and Vascular Center Hamburg, 20251 Hamburg, Germany; t.zeller@uke.de (T.Z.); christian_m@gmx.net (C.M.); d.boernigen@uke.de (D.B.); 4 Division of Anatomy & MIC, Medical University of Vienna, 1090 Vienna, Austria; wolfgang.weninger@meduniwien.ac.at (W.J.W.); stefan.geyer@meduniwien.ac.at (S.H.G.); 5 Department of Internal Medicine V, Hematology and Oncology, Medical University Innsbruck, 6020 Innsbruck, Austria; sieghart.sopper@i-med.ac.at; 6 Division of Bioinformatics, Medical University Innsbruck, Biocenter, 6020 Innsbruck, Austria; anne.krogsdam@i-med.ac.at

**Keywords:** CXCL12, cardiac hypertrophy, fibrosis, remodeling, M2 macrophages, coronary artery, smooth muscle cells

## Abstract

The chemokine CXCL12 plays a fundamental role in cardiovascular development, cell trafficking, and myocardial repair. Human genome-wide association studies even have identified novel loci downstream of the CXCL12 gene locus associated with coronary artery disease and myocardial infarction. Nevertheless, cell and tissue specific effects of CXCL12 are barely understood. Since we detected high expression of CXCL12 in smooth muscle (SM) cells, we generated a SM22-alpha-Cre driven mouse model to ablate CXCL12 (SM-CXCL12^−/−^). SM-CXCL12^−/−^ mice revealed high embryonic lethality (50%) with developmental defects, including aberrant topology of coronary arteries. Postnatally, SM-CXCL12^−/−^ mice developed severe cardiac hypertrophy associated with fibrosis, apoptotic cell death, impaired heart function, and severe coronary vascular defects characterized by thinned and dilated arteries. Transcriptome analyses showed specific upregulation of pathways associated with hypertrophic cardiomyopathy, collagen protein network, heart-related proteoglycans, and downregulation of the M2 macrophage modulators. CXCL12 mutants showed endothelial downregulation of the CXCL12 co-receptor CXCR7. Treatment of SM-CXCL12^−/−^ mice with the CXCR7 agonist TC14012 attenuated cardiac hypertrophy associated with increased pERK signaling. Our data suggest a critical role of smooth muscle-specific CXCL12 in arterial development, vessel maturation, and cardiac hypertrophy. Pharmacological stimulation of CXCR7 might be a promising target to attenuate adverse hypertrophic remodeling.

## 1. Introduction

Historically, CXCL12/SDF-1 is a CXC chemokine, which plays a crucial role in maintaining the bone marrow (BM) niche of hematopoietic stem and progenitor cells [1]. CXCL12 binding to its receptors CXCR4 and CXCR7 also plays an important role in cardiovascular development and is critically involved in leukocyte and progenitor cell trafficking to sites of myocardial ischemia [1,2,3]. The recently identified second CXCL12 receptor CXCR7 also plays a prominent role in cardiac valve morphogenesis and remodeling after myocardial infarction (MI) [4,5,6]. CXCL12 and CXCR4 interactions are also involved in tumor growth and metastasis by induction of angiogenesis and expression of CXCR4 on metastatic cancer cells [7]. Global CXCL12 as well as CXCR4 and CXCR7 knock-out (KO) mice reveal phenotypically ventricular septum defects and severe vascular abnormalities and die early perinatally [4,8,9,10,11]. During embryonic development, loci of vasculogenesis are characterized by high expression of CXCL12, CXCR4, and CXCR7 [4,12]. From a clinical translational perspective, human genome-wide association studies (GWAS) in over 100,000 people have identified two novel loci downstream of the CXCL12 gene locus associated with coronary artery disease (CAD) and MI, implicating an essential role in cardiovascular disease (CVD) [13].

Intramyocardial delivery of CXCL12 in phase 1 and phase 2 clinical trials in ischemic heart disease showed clinical improvement, suggesting therapeutic benefits of CXCL12 [14,15]. CXCL12 gene expression after ischemia is regulated by HIF-1α binding to the CXCL12 promoter with consecutive upregulation after acute MI for several days followed by a subsequent decline [16,17,18]. Invasive strategies to overexpress CXCL12 mRNA or deliver CXCL12 protein to the heart have been developed [16,19,20]. We have demonstrated previously that a dual non-invasive strategy based on the mobilization of progenitor cells with G-CSF and pharmacological inhibition of the CXCL12 inactivating protease DPP-IV/CD26 enhanced migration of CXCR4+ blood-derived progenitors and increased the number of endogenous lin-/c-kit+/Sca-1+ stem cells in the ischemic heart associated with decreased mortality and improved cardiac function in mice [17]. A combined strategy of G-CSF treatment with DPP-IV inhibition and cell cycle activation in cardiomyocytes by overexpression of cyclin D2 was even capable of enhancing myocardial regeneration after MI [21].

Although there is a clear potential to exploit the CXCL12/CXCR4/CXCR7 axis for therapeutic interventions, cell and tissue specific effects of CXCL12 in the cardiovascular system are barely understood, hindering the implementation of targeted therapies. The constitutive and inducible expression of this chemokine has been reported in several cell types of the heart like vascular endothelial cells, smooth muscle cells (SMCs), cardiomyocytes, fibroblasts, and pericytes [18,22,23,24]. So far, the underlying biology of these cell types regarding direct involvement in CXCL12-dependent cardiovascular development, cell recruitment, and myocardial repair mechanisms remains unclear. Additionally, a cardiomyocyte specific conditional CXCL12 KO mouse model does not display cardiovascular development defects, implicating divergent cellular functions [25]. Since our own preliminary data revealed high expression of CXCL12 in smooth muscle protein 22-alpha (SM22α) positive SMCs, we aimed to investigate the cell-specific role in the cardiovascular system by generating a SM22α-Cre driven conditional KO (cKO) mouse model (SM-CXCL12^−/−^).

## 2. Results

### 2.1. CXCL12 Is Predominantly Expressed in Vascular Smooth Muscle Cells

**Figure 1:**
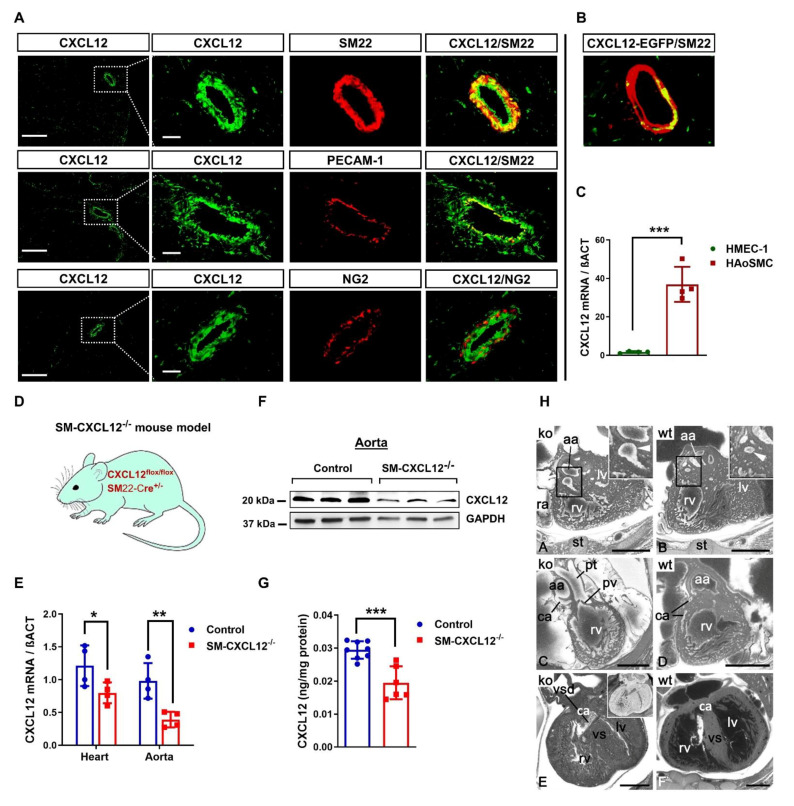
Conditional knockout (cKO) mouse model due to high expression of CXCL12 in vascular smooth muscle cells. (**A**) Immunofluorescence images of heart sections stained against CXCL12 (green) co-labeled with the smooth muscle cell specific marker SM22-alpha (SM22) (red; upper row), the endothelial cell marker PECAM-1 (red, middle row), and the pericyte marker NG2 (red, lower row). Scale bar, 200 and 25 μm. (**B**) Immunostaining of heart sections from CXCL12-EGFP reporter mice co-stained with SM22. Scale bar, 25 μm. (**C**) Bar graph representing the quantification of SDF-1 mRNA expression in human HAoSMC and HMEC-1 cells, *n* = 4. *** *p* < 0.001 from Student’s *t*-test (**D**) Schematic illustration of the smooth muscle cell-specific conditional KO mouse model. (**E**) Real-time PCR quantification of mRNA derived from heart and aorta of control (flox/flox) and SM-SDF-1^−/−^ mice, *n* = 4. * *p* < 0.05, ** *p* < 0.01 from two-way ANOVA followed by Sidak’s multiple comparisons test. (**F**) Western blot of SDF-1 protein from aortic tissue of mice related to GAPDH as endogenous reference protein, confirming reduced SDF-1 expression, *n* = 3. (**G**) ELISA of plasma SDF-1 levels quantified and related to total protein concentration in both control (*n* = 8) and mutant mice (*n* = 6). *** *p* < 0.001 from Student’s *t*-test. (**H**) Axial HREM section through the heart from cranial. A–D. Abnormal coronary arteries (ca) at E14.5. A. Dilated right coronary artery (arrowhead in inlay). C. Abnormal origin and topology of left coronary artery. B, D. controls. E, F. Muscular ventricular septal defect (vsd) at E17.5. Inlay in E shows axially sectioned volume model from cranial. F. control. aa, ascending aorta; ra, right atrial appendix; st, sternum; rv, right ventricle; lv, left ventricle; vs, ventricular septum; pv, pulmonary valve; pt, pulmonary trunk. Scale bars 500 µm. All data represent mean ± SD.

Since the expression pattern of CXCL12 in adult heart tissue is not well examined, we first aimed to characterize cellular sources of CXCL12 using specific antibodies staining the CXCL12 epitope. As shown in Figure 1A (first lane), CXCL12 expression predominantly targeted to vascular SMCs confirmed by co-staining of CXCL12 with an antibody specific to the SM22α epitope in SMCs. In contrast, only moderate or low expression was found in endothelial (PECAM-1) and perivascular cells (NG2) (Figure 1A middle lane and lower lane). Our results were confirmed on the expression level using CXCL12-EGFP reporter mice showing a predominant expression of CXCL12 in SMCs (Figure 1B). Translationally, CXCL12 was also highly expressed in human aortic smooth muscle cells (HAoSMC) displaying a ≥20-fold higher expression compared to human microvascular endothelial cells (HMEC-1), suggesting that SMCs are also a major cellular source for CXCL12 in humans (Figure 1C).

### 2.2. Loss of CXCL12 in SMCs Conferred Substantial Perinatal Mortality and Cardiovascular Abnormalities

Since CXCL12 expression was mainly targeted to SM22α positive SMCs, we generated a conditional knockout (SM-CXCL12^−/−^) mouse model of CXCL12 in SMCs by crossing CXCL12 flox with SM22α-Cre^+^ mice (Figure 1D). Deletion of the CXCL12 exon 1 was confirmed by PCR genotyping, as shown in Supplementary Figure S1A. Loss of CXCL12 in SMCs led to a significant downregulation of CXCL12 mRNA and protein in the heart and aorta (Figure 1E,F). We also noticed a 30–40% decrease in plasma CXCL12 levels in SM-CXCL12^−/−^ mice (Figure 1G). We found no significant changes in mRNA expression levels of the CXCL12 receptors CXCR4 and CXCR7 in mutant mice vs. controls (Supplementary Figure S1B). SM-CXCL12^−/−^ mice displayed embryonic growth retardation (Supplementary Figure S1C). Offsprings exhibited an abnormal mendelian ratio with ca. 50% perinatal mortality (Supplementary Figure S1D). High-resolution episcopic microscopy analysis (HREM) of CXCL12 deficient embryos revealed severe cardiovascular abnormalities. These included abnormal origin, abnormal topology, and dilation of segments of coronary arteries (A&C), abnormal dimensions of segments of the head arteries, bicuspid aortic valves (data not shown), defective cusps of semilunar valves (C), ventricular septal defects (E), and enlarged liver sinusoids (data not shown) (Figure 1H).

### 2.3. Smooth Muscle-Specific CXCL12 KO Mutants Developed Severe Cardiac Hypertrophy

**Figure 2:**
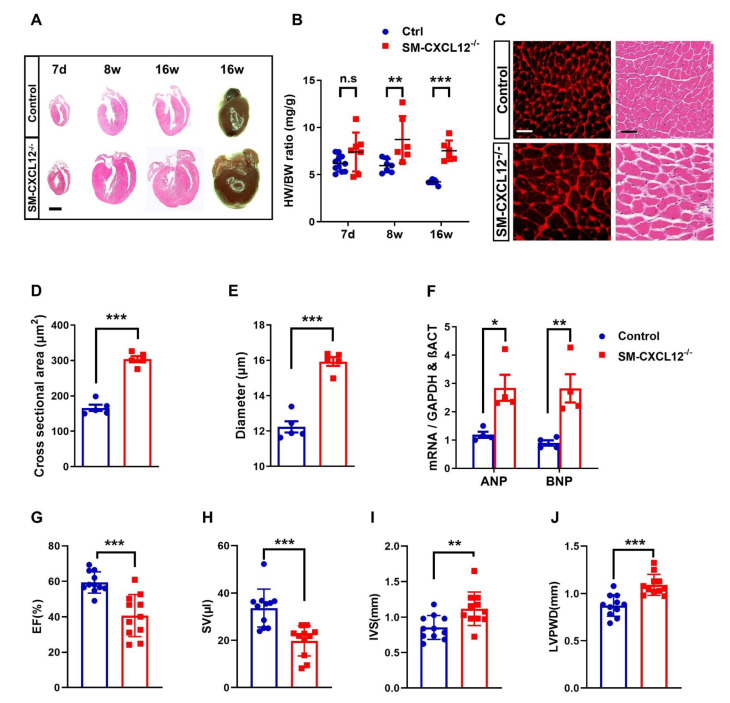
Cardiac hypertrophy and ventricular dysfunction in SM-CXCL12^−/−^ mice. (**A**) H&E stained heart sections at 7 days (7 d), 8 weeks (8 w), and 16 weeks (16 w) after birth as well as stereo images of whole hearts from 16 weeks (16 w) control and cKO mice. Scale bar, 2 mm. (**B**) Heart weight/body weight (HW/BW) ratios (mg/g) of control and cKO mice at various time points (7 d, 8 w, 16 w), *n* = 6–11. n.s. not significant, ** *p* < 0.01, *** *p* < 0.001 from two-way ANOVA followed by Sidak’s multiple comparisons test. (**C**) Immunofluorescence staining of wheat germ agglutinin (WGA; red, first row) and H&E (second row) of left ventricular heart sections in control and cKO mice. Scale bar, 20 µm. (**D**,**E**) Bar graphs representing the cross-sectional area and minimum Feret’s diameter of adult cardiomyocytes, *n* = 5. *** *p* < 0.001 from Student’s *t*-test (**F**) qRT-PCR analysis of the cardiac hypertrophy markers ANP and BNP mRNA relative to the expression of the housekeeping genes GAPDH and β-Actin, *n* = 4. * *p* < 0.05, ** *p* < 0.01 from two-way ANOVA followed by Sidak’s multiple comparisons test. (**G**–**J**) Measurements of ejection fraction (EF), stroke volume (SV), interventricular septum thickness (IVS), left ventricular posterior wall diameter (LVPWD) of control and cKO mice, *n* = 11. ** *p* < 0.01, *** *p* < 0.001 from Student’s *t*-test. All data are presented as mean ± SD.

Surviving SM-CXCL12^−/−^ mice developed an age-dependent cardiac hypertrophy after birth. While there was a trend to an increased heart weight to body weight ratio at seven days after birth, the ratio significantly increased during adolescence at eight and 16 weeks of age (Figure 2A,B). 

Histology of adult hearts stained with H&E and WGA confirmed hypertrophy of cardiomyocytes in cKO mice (Figure 2C). Cardiomyocyte cross-sectional areas and diameters were significantly increased in mutant hearts as compared to controls (Figure 2D,E). The development of cardiac hypertrophy was further confirmed by quantification of typical hypertrophy markers, such as atrial natriuretic peptide (ANP) and brain natriuretic peptide (BNP) in cardiac tissues. Accordingly, ANP and BNP mRNA levels were significantly upregulated in mutant mice (Figure 2F). Echocardiography measurements of adult hearts at 20 weeks also revealed significant differences in left ventricular functional parameters such as decreased ejection fraction, fractional shortening, and stroke volume and a significant increase in the thickness of the interventricular septum and left ventricular posterior wall diameters, confirming cardiac hypertrophy (Figure 2G–J, Supplementary Table S1.

### 2.4. SM-CXCL12^−/−^ Hearts Revealed Increased Cardiac Fibrosis and Abnormal Coronary Arteries

**Figure 3:**
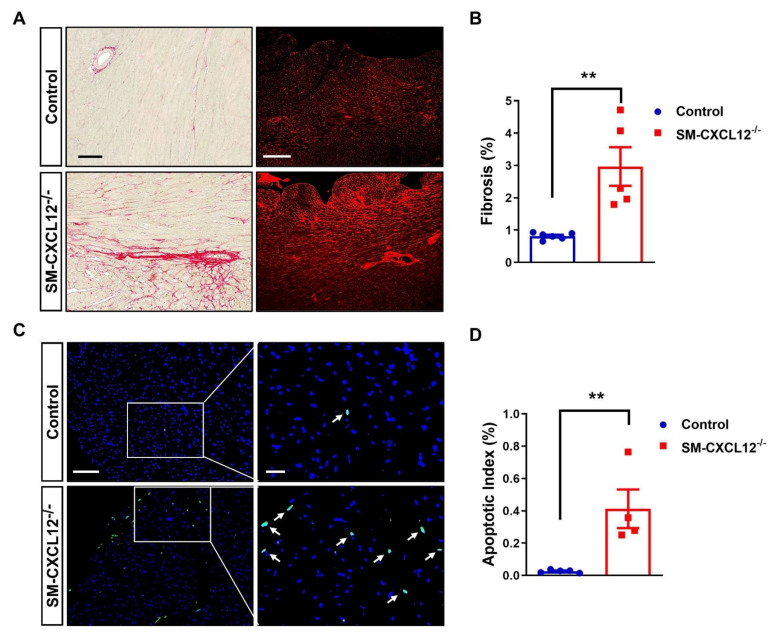
SM-CXCL12^−/−^ mice revealed increased cardiac fibrosis, apoptosis. (**A**) Representative Sirius red and WGA stainings of heart sections from WT and cKO mice (left panel), scale bar represents 100 and 200 µm. (**B**) Bar graph showing quantification of the ratio of myocardial fibrosis area to total myocardial area in histological sections (right panel; *n* = 5–6), ** *p* < 0.01 from Student’s *t*-test (**C**) Representative co-staining of TUNEL+ (bright green nuclei marked by arrows) and DAPI+ (blue nuclei) cells in WT and cKO mice (left panel), Scale bar represents 100 and 25 µm. (**D**) Bar graph showing quantification of the percentage of TUNEL+ apoptotic nuclei to total nuclei (right panel; *n* = 4–5), ** *p* < 0.01 from Student’s *t*-test. Data represent mean ± SD.

**Figure 4:**
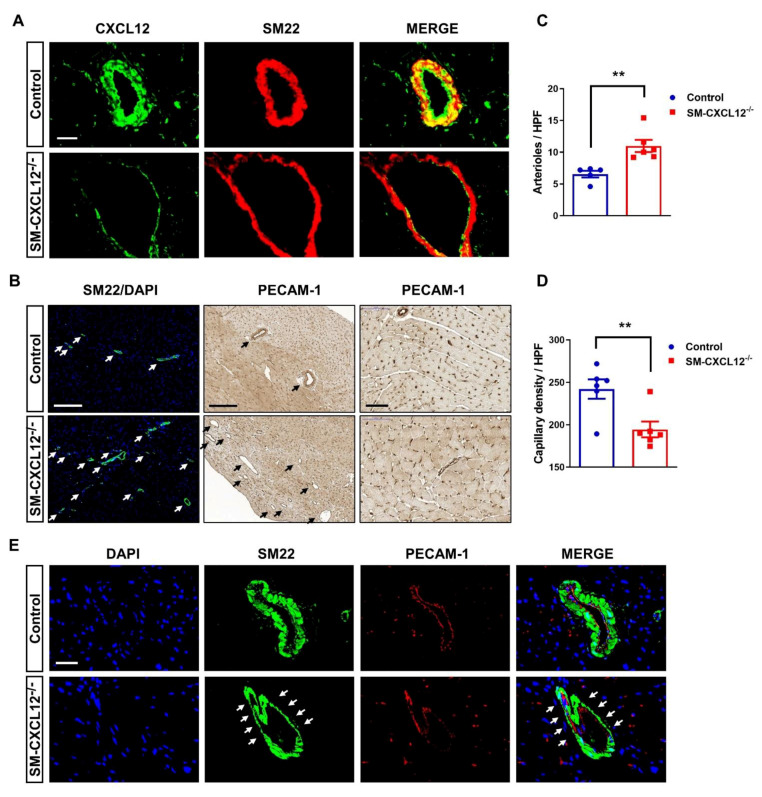
SM22α-specific ablation of CXCL12 leads to defective coronary arteries. (**A**) Co-staining of CXCL12 (green) and SM22 (red) in arteries of control and SM-CXCL12^−/−^ mice. (**B**) First column: SM22/DAPI immunofluorescence staining of heart sections from control and SM-CXCL12^−/−^ mice showing an increased density of arteries and arterioles in cKO mice. Second column: immunostaining of PECAM-1 positive (brown) capillaries and arterioles at lower magnification (10×) showing an increased density of aberrant arteries and arterioles (depicted by black arrows) in cKO mice. Third column: immunostaining showing a decreased density of PECAM-1 positive capillaries in cKO mice. Scale bar, 200 and 20 µm. (**C**,**D**) Bar graphs showing the quantification of arterioles and capillary density in control and SM-CXCL12^−/−^ hearts, *n* = 5 and 5 independent fields per each mouse. ** *p* < 0.01, from Student’s *t*-test (**E**) SM22/PECAM-1/DAPI immunofluorescence staining of control and SM-SDF-1^−/−^ heart sections at higher magnification (40×) showing a defective smooth muscle cell layer (depicted by white arrows) characterized by dilated and thinned arteries in cKO mice. Scale bar, 20 µm. All data are presented as mean ± SD.

Next, we examined adult hearts histologically. Sirius red and WGA staining revealed an increased amount of interstitial and perivascular cardiac fibrosis in hypertrophic KO hearts (Figure 3A,B). TUNEL+ staining displayed a significant increase in apoptotic cell death (Figure 3C,D). It is noteworthy to mention that we also detected a slight increase in pH3+ cardiomyocytes in KO hearts suggesting at least some degree of hyperplasia preceding the hypertrophic response (Supplementary Figure S2A,B). Further immunofluorescence staining of mutant hearts confirmed a lack of CXCL12 expression in vascular smooth muscle cells; however, we still observed low amounts of CXCL12 expression in endothelial cells (Figure 4A). Since complete loss of CXCL12 plays a crucial role in arterial development, maturation, and patterning of the aortic arch [26,27], we investigated arterial and capillary density in KO hearts using anti-SM22α and anti-PECAM-1 specific antibodies (Figure 4B). We observed an increase in dilated SM22α positive aberrant arteries in cKO hearts, whereas capillary density was decreased, most likely due to cardiac hypertrophy (Figure 4C,D). However, despite an increase in arteriole density, mutant mice exhibited malformed, thinned, and dilated arteries with almost no distinguishable SMC layer (Figure 4A,E).

### 2.5. RNAseq Analysis Revealed Increased Signaling for Hypertrophic Cardiomyopathy and Downregulation of M2 Macrophage Markers RETNLA and CXCL14

**Figure 5:**
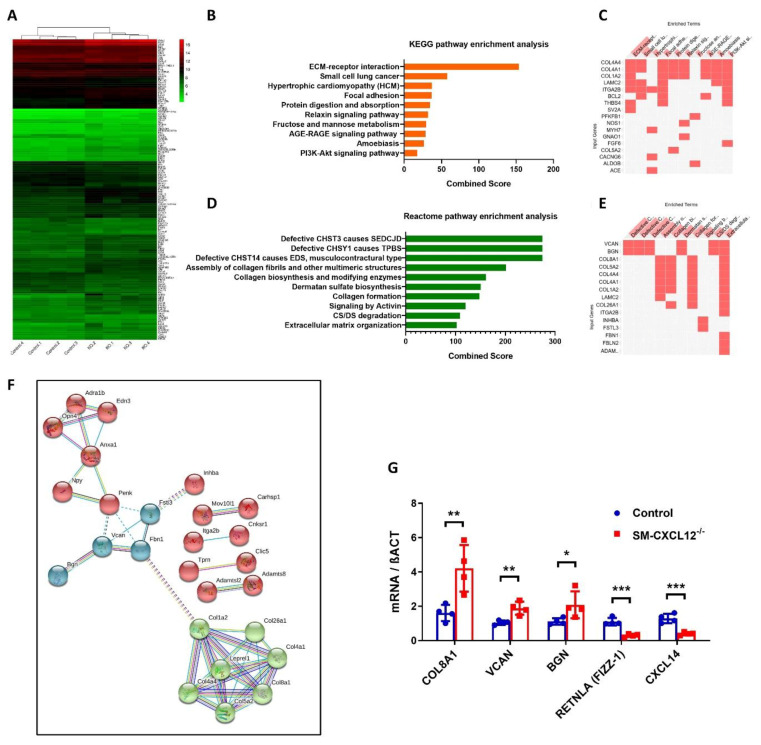
Cardiac transcriptome analysis of SM-CXCL12^−/−^ mice. (**A**) Hierarchically clustered heatmap showing 143 differentially expressed genes (DEGs) in the hearts of SM-CXCL12^−/−^ mice relative to controls, *n* = 4 biological replicates. Fold change > 1.5, *p* < 0.05. (**B**,**D**) Bar graph visualization of top 10 Kyoto Encyclopedia of Genes and Genomes (KEGG) and Reactome pathway enrichment analyses of DEGs using Enrichr. (**C**,**E**) Clustergram displaying the heatmap of top 10 enriched terms (KEGG and Reactome pathways as columns) vs. input genes (as rows). (**F**) STRING protein–protein interaction (PPI) networks of significantly regulated genes. Each node represents a distinct color with network clustered (kmeans) and disconnected nodes hidden from the network display. Line color indicates the type of interaction evidence. (**G**) Validation of the differentially expressed genes COL8A1, VCAN, BGN, RETNLA, and CXCL14 by qRT-PCR, *n* = 4. * *p* < 0.05, ** *p* < 0.01, *** *p* < 0.001 from two-way ANOVA followed by Sidak’s multiple comparisons test. Data are mean ± SD.

To gain more mechanistic insights into the cellular pathways, whole transcriptome analyses were performed to compare differentially expressed genes between control and conditional KO hearts. We identified 143 differentially expressed genes (DEGs) with expression differences of more than 1.5-fold, and a *p*-value < 0.05. Ninety-three genes were significantly upregulated, and 50 genes were significantly downregulated. The heat map analysis of DEGs is depicted in Figure 5A and listed in Supplementary Table S2. Amongst downregulated transcripts, we detected the M2 macrophage associated genes RETNLA (FIZZ-1) and CXCL14. Next, we performed pathway analysis of DEGs using the pathway enrichment analysis tool enrichR (https://maayanlab.cloud/Enrichr/enrich?dataset=9ae3742d8fd7ee20209051133e10491c, access date 30 May 2021). KEGG pathway enrichment analysis with DEGs highly clustered in several signaling pathways including extracellular matrix receptor interaction, hypertrophic cardiomyopathy, focal adhesion, and PI3K-Akt signaling. The top 10 enriched pathways are shown in Figure 5B,C and listed in the Supplementary Table S3. Similarly, reactome pathway enrichment analysis showed that clustered signaling pathways were related to an extracellular matrix organization, collagen biosynthesis and degradation. Additionally, chondroitin sulfate (CS) and dermatan sulfate (DS) proteoglycan pathways were significantly upregulated (Figure 5D,E, Supplementary Table S4). We further predicted the protein–protein interaction network of DEGs using STRING web software and observed two major protein clusters that closely interacted with each other. The green-colored clustered protein network was specific to collagen regulation, whereas the blue-colored clustered proteins were involved in proteoglycan synthesis and degradation (VCAN & BGN) (Figure 5F). Finally, we validated transcriptome data of DEGs with qPCR analysis, confirming upregulation of genes such as COL8A1, VCAN, and BGN and downregulation of the M2 macrophage associated genes RETNLA and CXCL14 in KO hearts (Figure 5G).

### 2.6. SM-CXCL12^−/−^ Mouse Hearts Showed Decreased M2-Like Macrophages (CD206 + )

**Figure 6:**
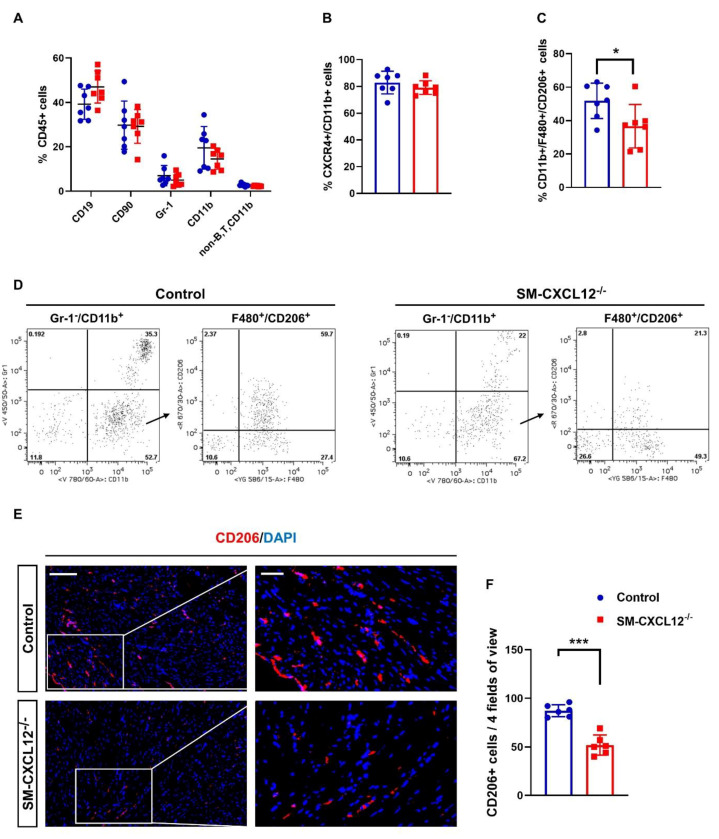
Cardiac M2-like macrophages were declined in SM-CXCL12^−/−^ mice. (**A**) Bar graph showing flow cytometric analysis of CD45+ leukocyte subsets gated and quantified for CD19+ B lymphocytes, CD90+ T lymphocytes, Gr-1 granulocytes, CD11b monocytes/macrophages, *n* = 7. (**B**) Bar graph showing the percentage of CXCR4 + /CD11b+ cells in the hearts of control and cKO mice, *n* = 7. (**C**) Quantification of cardiac Gr-1-/CD11b+/F480+/CD206+ cells showing a significant reduction in SM-CXCL12^−/−^ mice, *n* = 7. * *p* < 0.05 from Student’s *t*-test. (**D**) Representative gating strategy and scatter plots used to identify M2-like macrophage populations (Gr-1-/CD11b+/F480+/CD206+): For quantification of M2 macrophages, granulocyte negative CD11b+ cells were gated and stained for F480 and CD206. (**E**) Immunofluorescence labeling against CD206/DAPI confirmed a significant reduction of CD206+ cells in the myocardium of cKO mice. (**F**) Bar graph representing the quantification of cardiac CD206+ M2 like cells in the hearts of control and mutant mice, *n* = 6. Scale bar, 200 µm. *** *p* < 0.001 from Student’s *t*-test. Data represent mean ± SD.

Since CXCL12 plays a major role in activating and recruiting of leukocytes, flow cytometry analyses of BM, spleens and hearts of SM-CXCL12^−/−^ mice, and littermate controls were performed to assess the immune cell response. A panel of different leukocyte markers such as CD19+ (B-lymphocytes), CD90+ (T-lymphocytes), Gr-1+ (granulocyte), CD11b+ (monocyte/macrophage), CD206+ (M2-macrophage), and CD184+ (CXCR4+) was used to screen for subsets of CD45+ expressing cells (Figure 6A).

We did not observe any significant differences in BM and spleen leukocyte cell populations between mutants and controls (Supplementary Figure S3A–H). Cardiac CD45+ leukocyte subpopulations including CD45+/CD11b+ and CXCR4+/CD11b+ cells were also not significantly different (Figure 6A,B). However, SM-CXCL12^−/−^ mouse hearts showed a significant depletion of M2 like Gr-1-/CD11b+/F480+/CD206+ cells (Figure 6C,D). Immunofluorescence analysis of SM-CXCL12^−/−^ mice hearts further confirmed the finding of a significant reduction of M2 like CD206+ cells (Figure 6E,F).

### 2.7. Downregulation of the CXCL12 Co-Receptor CXCR7 in SM-CXCL12^−/−^ Hearts

**Figure 7:**
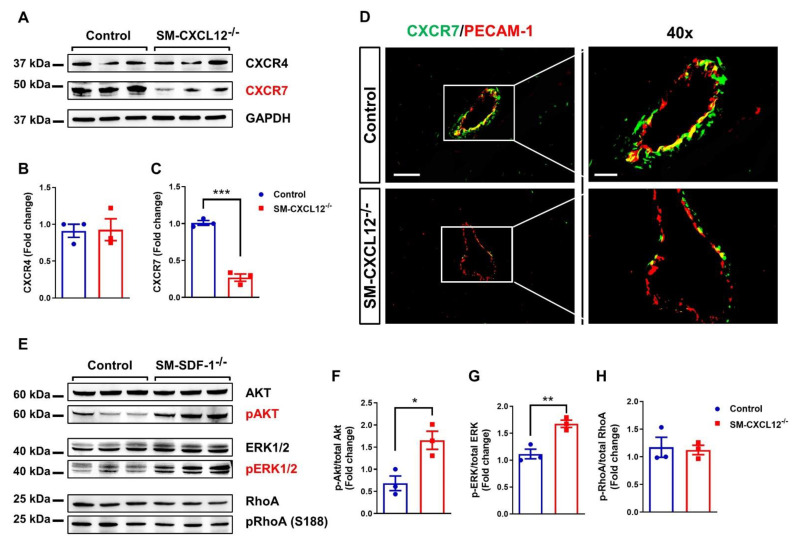
Decreased CXCR7 expression and activation of AKT and ERK1/2 signaling pathways in hypertrophic cKO hearts. (**A**–**C**) Representative Western blot analysis and bar graphs showing the quantification of CXCR4 and CXCR7 protein levels in heart lysates of control and cKO mice. *** *p* < 0.001 from Student’s *t*-test. GAPDH was used as reference protein, *n* = 3. (**D**) Heart sections of mice immunolabeled with antibodies against CXCR7 (green) and the endothelial cell marker PECAM-1 (red). Scale bar, 200 and 20 µm. (**E**–**H**) Western blot analyses and bar graphs represent the quantification of phosphorylated and total protein levels of AKT, ERK1/2, and RhoA in mouse hearts of control and SM-CXCL12^−/−^ mice, *n* = 3. * *p* < 0.05, ** *p* < 0.01 from Student’s *t*-test. Data represent mean ± SD.

Since CXCL12 binds to its corresponding receptors CXCR4 and CXCR7 and activates multiple signaling pathways, we analyzed receptor protein expression in cKO hearts. While CXCR4 protein levels were unchanged, similar to mRNA expression data, we observed a significant decrease of CXCR7 protein in mutant hearts (Figure 7A–C). Furthermore, we histologically examined CXCR4 and CXCR7 expression in heart tissue. CXCR4 expression was strongly detected in both smooth muscle and endothelial cells of arteries in control hearts, confirmed by co-expression of the endothelial cell marker PECAM-1 (Supplementary Figure S4). Likewise, Western blot and immunofluorescence analyses confirmed that expression of CXCR4 was not altered in SM-CXCL12^−/−^ mice. On the other hand, CXCR7 expression was mainly localized in endothelial cells of coronary arteries in control mice. SM-CXCL12^−/−^ mutants revealed a highly decreased amount of endothelial CXCR7 expression in coronary arteries (Figure 7D). We further examined key downstream signaling pathways of CXCL12 such as Akt, ERK, and RhoA. As shown in Figure 7E–H, we detected increased phosphorylation of Akt and ERK in KO hearts, whereas RhoA levels remained unchanged.

### 2.8. CXCR7 Agonist Treatment Attenuated Hypertrophic Remodeling in SM-CXCL12^−/−^ Mice Associated with Activation of pERK

**Figure 8:**
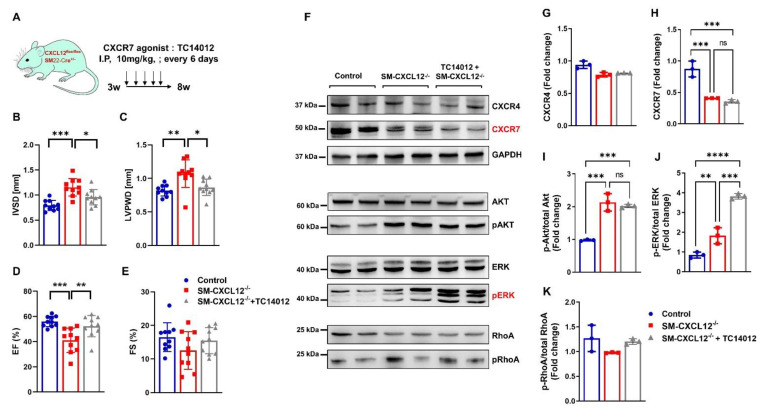
Treatment with the CXCR7 agonist TC14012 attenuated hypertrophic remodeling and activated pERK signaling in SM-CXCL12^−/−^ mice. (**A**) Experimental scheme studying the effects of the CXCR7 agonist TC14012 in SM-CXCL12^−/−^ mice. (**B**–**E**) Bar graphs representing the echocardiographic parameters (**B**) interventricular septum diameter (IVSD), (**C**) left ventricular posterior wall diameter (LVPWD), (**D**) ejection fraction (EF), and (**E**) fractional shortening (FS), *n* = 10 per group. * *p* < 0.05, ** *p* < 0.01, *** *p* < 0.001, **** *p* < 0.0001 from one-way ANOVA followed by Tukey’s multiple comparisons test. (**F**) Western blot analyses of CXCR4, CXCR7, and GAPDH, phosphorylated and total protein levels of AKT, ERK1/2, and RhoA in heart lysates of controls, SM-CXCL12^−/−^, and cKO + TC14012 mice. (**G**–**K**) Bar graphs displaying the quantification of CXCR4, CXCR7 expression, and phosphorylated protein levels of AKT, ERK1/2, and RhoA, *n* = 3, ** *p* < 0.01, *** *p* < 0.001, **** *p* < 0.0001 from one-way ANOVA followed by Tukey’s multiple comparisons test. Data represent mean ± SD.

Given that both CXCL12 and CXCR7 were significantly reduced in SM-CXCL12^−/−^ mice, we sought to explore potential benefits by specifically activating the CXCL12/CXCR7 axis in SM-CXCL12^−/−^ mice. Consequently, we administered the specific CXCR7 agonist TC14012 intraperitoneally over 5 weeks as a gain of function experiment (Figure 8A, Supplementary Table S5). As shown in Figure 8B,C, SM-CXCL12^−/−^ mice treated with TC14012 showed significantly reduced interventricular septum (IVSD) and left ventricular posterior wall diameters (LVPWD) reflecting attenuated progression of cardiac hypertrophy. Moreover, CXCR7 agonistic treatment showed an improved left ventricular ejection fraction (Figure 8D,E, Supplementary Table S5). There was also a tendency towards decreased left ventricular end-diastolic and systolic diameters after the treatment (Supplementary Table S5). Finally, we investigated potential CXCR7 downstream signaling pathways like Akt, ERK, and RhoA. As shown before in Figure 7E–H, we observed increased phosphorylation of Akt and ERK signaling in cKO mice hearts and further noticed a markedly specific activation of pERK signaling after CXCR7 agonist treatment (Figure 8F–K). Our data suggest that CXCR7 agonistic treatment acts through pERK signaling to attenuate progression of cardiac hypertrophy.

## 3. Discussion

The chemokine CXCL12 plays an important role in cell migration, differentiation, tissue homeostasis of leukocytes and hematopoietic stem and progenitor cells, and is critically involved in ischemic tissue repair [3,16,20,22]. However, cell- and tissue-specific effects of CXCL12 are barely understood, limiting the implementation of targeted therapies. Here, we show for the first time a prominent role of SMC derived CXCL12 for vessel development and maturation, progression of cardiac hypertrophy, and tissue homeostasis of CD206 macrophages. On the regulatory level, cKO of CXCL12 in SMCs led to decreased expression of its corresponding receptor CXCR7 on endothelial cells, whereas expression of the common CXCL12 receptor CXCR4 remained unchanged. Treatment with the CXCR7 agonist TC14012 attenuated progression of cardiac hypertrophy and restored cardiac function associated with increased activation of pERK signaling.

CXCL12-EGFP reporter mice and immunofluorescence staining against SM22α demonstrated high expression of CXCL12 on the transcriptional and protein level in SMCs of arterial vessels, whereas pericytes and endothelial cells expressed CXCL12 to a much lower extent. Translationally, we also found high expression of CXCL12 in human aortic smooth muscle cells compared to human microvascular endothelial cells. Based on these findings, we aimed to investigate the cell-specific role of CXCL12 in SMCs utilizing a SM22α-Cre driven CXCL12 mutant mouse model. In this study, we have deliberately chosen the widely used SM22α-Cre model (also known as Tagln-cre) to drive Cre expression in smooth muscle cells, since we clearly detected CXCL12 in SM22α positive arterial SMCs [28,29,30]. Other possible SMC-targeting Cre mouse models such as Myh11-Cre or Acta2-Cre might have the caveat that Myh11-Cre is also expressed in pericytes [31], and Acta2-Cre was found to be expressed in other cell types like fibroblasts and myofibroblasts [31]. The importance of SMCs as a relevant source of circulating CXCL12 was confirmed by a 30–40% decrease in CXCL12 plasma levels compared to control mice. SM-CXCL12^−/−^ mice revealed a high embryonic lethality (50%) and displayed developmental defects including abnormal origin and topology of coronary arteries and ventricular septum defects in line with previous findings from complete CXCL12 KO mice, complementing these data by specifying the importance of SMC derived CXCL12 in cardiovascular development for the first time.

Our cKO model revealed enlarged defective coronary arteries with pronounced thinning of the SMC media layer, suggesting a very important role of SMC-derived CXCL12 for coronary artery integrity and maturation. In line with our findings are two recently published studies showing defective coronary artery development and lack of SM22 specific vessels in constitutive CXCL12 KO mice, also illustrating the importance of CXCL12 for the formation and integrity of coronary arteries [26,27]. However, our data extends these reports, revealing SMC-derived CXCL12 as a major cellular source for coronary artery defects. Moreover, the clinical translationally importance of CXCL12 in the human coronary artery system is substantiated by various genome-wide association studies, showing that single nucleotide polymorphisms (SNPs) close to the human CXCL12 locus were linked to CAD and MI. These risk alleles were associated with increased plasma levels of CXCL12 [32,33,34,35,36]. Additionally, global CXCL12 and CXCR4 KO mouse embryos also displayed defects in vascularization of the gastrointestinal tract and organ-specific processes of arteriogenesis [10,37]. A previous study showed that prolonged delivery of protease-resistant CXCL12 increased blood flow and arteriolar density in a model of hindlimb ischemia, suggesting that CXCL12 might be important for arteriogenesis [38].

Another important new finding of our study is that SM-CXCL12^−/−^ mice developed severe cardiac hypertrophy associated with fibrosis and apoptosis. It is known that therapeutic targeting of CXCL12 during ischemia leads to cardiac protection through decreased fibrosis and apoptosis [17,18,19,39]. However, there is only limited evidence showing a substantial role of CXCL12 in the progression of cardiac hypertrophy. In our model, lack of SMC-derived CXCL12 led to severe cardiac hypertrophy associated with progressive fibrosis and apoptosis. Supporting our data, a recently published study showed that cardiomyocyte specific deletion of the CXCL12 corresponding receptor CXCR4 in mice leads to progressive cardiomyopathy with significant tissue fibrosis [40]. In this model, isoproterenol-induced cardiac hypertrophy was related to worsening of cardiac function, increased fibrosis, and apoptosis, implicating CXCL12/CXCR4 signaling in the regulation of cardiac hypertrophy, tissue remodeling, and apoptosis [41]. The second corresponding receptor for CXCL12, CXCR7, is also known to be involved in cardiac hypertrophy. Specifically, endothelial specific deletion of CXCR7 revealed a marked cardiac hypertrophy and displayed a key role in cardiac remodeling after MI [4,5,6]. As a link to human pathology, two studies in human subjects suffering from hypertrophic cardiomyopathy (HCM) showed elevated plasma levels of CXCL12 related to increased diffuse fibrosis, also suggesting an important role of CXCL12 in human pathology [42,43]. Therefore, our data may provide a background for a more detailed evaluation of the impact of CXCL12 in HCM in future studies.

CXCL12 displays a central role in hematopoiesis and BM myelopoiesis during embryonic development, as well as leukocyte and progenitor cell trafficking in organ repair [1,8]. Flowcytometry analyses of BM and spleens of our SM-CXCL12^−/−^ mice revealed no significant differences in several leukocyte populations. Interestingly, we observed a prominent decrease in tissue-resident M2-like CD11b+/F480+/CD206+ macrophages in mutant hearts. In addition, immunostaining confirmed that cardiac CD206+ cells were markedly reduced in SM-CXCL12^−/−^ mice. These findings are in line with our transcriptome data showing that Retnla, a hallmark of alternatively activated M2 macrophages, was significantly downregulated in cKO mice [44]. Moreover, CXCL14, a chemokine that has been recently described as inhibiting M1 macrophage polarization but increasing M2 polarization, was markedly reduced in SM-CXCL12^−/−^ mice, supporting a proinflammatory state [45]. Previous studies have shown that cardiac M2-like macrophages exhibit tremendous anti-inflammatory and tissue repair capabilities, whereas M1-like macrophages rather reflect a pro-inflammatory status after MI [44,46,47]. Additionally, macrophages also contribute to tissue fibrosis and homeostasis [48]. M1 macrophages are involved in the initiation of pro-fibrotic processes, whereas tissue resident M2 macrophages regulate fibrosis and control tissue repair and homeostasis [49]. On the other hand, it has been shown that depolarizing M2 to M1 macrophages can induce apoptosis in tumor cells, implicating a crucial role of M2 macrophages in regulation of apoptosis [50]. Our own previous data showed that HIF-1α mediated upregulation of CXCL12 by prolyl-hydroxylase inhibition increased reparative M2-like CD11b+/CD206+ subpopulations compared to M1-like CD11b+/CD86+ cells after MI associated with increased cardiac repair [51]. Collectively, our data suggest a specific role of SMC-derived CXCL12 in maintaining and differentiation of reparative M2-like cardiac macrophages in myocardial development and repair.

Since CXCL12 is the ligand for both the CXCR4 and CXCR7 receptor, we next examined their expression in cKO and control hearts. Mechanistically, we found expression of the commonly known G-protein coupled receptor CXCR4 on the transcriptional and protein level largely unchanged, whereas CXCR7 protein levels were consistently downregulated, arguing for an important role of CXCL12/CXCR7 for vessel maturation and progression of hypertrophic remodeling. CXCR7 expression was mainly targeted to endothelial cells of coronary arteries. Consistently, cKO of CXCR7 in ECs also showed a severe cardiac hypertrophy [4,5]. Since CXCR7 is a known scavenger receptor for CXCL12, leading to internalization and degradation, it could be hypothesized that CXCR7 regulates CXCL12/CXCR4 interactions through sequestering CXCL12 during heart development and therefore might be important for fine tuning of hematopoietic cell mobilization [52]. CXCR7 can also be internalized without ligand binding [53], suggesting that the observed downregulation of CXCR7 protein without changes in mRNA levels may reflect internalization and degradation of CXCR7 without ligand binding to maintain the homeostatic CXCL12 function. On the other hand, CXCR7 signaling is known to be transduced G-protein independent through β-arrestin mediated ERK phosphorylation [54,55]. Alternatively, CXCR7 can also form heterodimers with the CXCR4 receptor regulating G-protein-mediated signal transduction [4,56]. A recent study has shown that CXCR7 expression is elevated in human heart failure and hypothesized that it might have cardioprotective effects [57]. Mechanistically the authors also demonstrated that CXCR7 acts through β-arrestin-mediated pERK signaling. Several preclinical animal models have proven that CXCR7 activation has a protective role in acute MI and atherosclerotic vascular diseases [6,57,58]. To reactivate CXCR7 signaling, we treated our cKO mice with the CXCR7 agonist TC14012, which attenuated the severe hypertrophic phenotype and improved heart function (see also Figure 7). As a potential downstream target for CXCR7 signaling, we identified increased pERK signaling, which has also been reported previously [54,57,59]. CXCL12 signaling directly via CXCR7 is Gαi-receptor-independent, and activation of pERK could lead to cell survival and chemotaxis [60]. A recent study also reported downregulation of CXCR7 and pERK signaling in endothelial outgrowth cells derived from patients with coronary artery disease. Activation of CXCR7/pERK signaling increased vasculogenesis, suggesting a direct signaling effect of the CXCL12/CXCR7 axis [55]. Additionally, recent studies also demonstrated that agonizing CXCR7 contributed to therapeutic benefits in pulmonary fibrosis and acute MI, arguing for the importance of CXCL12/CXCR7 signaling [6,61]. Since the CXCR7 agonist TC14012 can also act as a CXCR4 antagonist [59], one of the major limitations of our study is that we cannot rule out the possibility that at least some beneficial effects after TC14012 treatment might be explained through CXCL12/CXCR4 inhibition. There is mounting evidence suggesting that antagonism of CXCR4 attenuates cardiac fibrosis and improves myocardial function in various heart failure models [59,62,63]. The opposite effects of TC14012 on CXCR4 and CXCR7 at the mechanistic level are still not well understood and need to be clarified in future studies. Additionally, even the structurally unrelated CXCR4 inhibitor AMD3100 also displays weak agonistic CXCR7 function, suggesting cross reactivity of these compounds on CXCR4 and CXCR7 receptors [59]. However, since TC14012 is a much more potent agonist on CXCR7 and treatment of cKO mice revealed a clear upregulation of pERK, which could not be explained through CXCR4 antagonism, our data suggest that CXCL12-CXCR7 mediated β-arrestin signaling plays an important role in attenuation of cardiac hypertrophy and remodeling. To date, this is the first study demonstrating potential clinical benefits for a CXCR7 agonist to limit the progression of cardiac hypertrophic remodeling. Although our study may be hypothesis building, further studies investigating the exact role of CXCL12/CXCR7 signaling in hypertrophic and vascular remodeling are warranted.

In summary, we provide completely novel data signifying the cell-specific role of smooth muscle-derived CXCL12 in cardiovascular development, arterial maturation, and progression to cardiac hypertrophy. Our data suggest that CXCL12 is critically involved in maintaining vascular homeostasis by regulating CXCR7 signaling. Pharmacological activation of CXCR7 might be a future target to attenuate excessive hypertrophic remodeling. Our findings could directly impact the development of treatments for patients with cardiac hypertrophy and CAD.

## 4. Materials and Methods

### 4.1. Mouse Strains

Animal care and all experimental procedures were performed in strict accordance with the Austrian animal legislation guidelines and conform to the guidelines from Directive 2010/63/EU of the European Parliament on the protection of animals used for scientific purposes. CXCL12 EGFP BAC reporter mice were purchased from Mutant Mouse Resource and Research Center (MMRRC). CXCL12^flox/flox^ mice were kindly gifted by Prof Michael Bader, MDC, Berlin, and Sm22a^cre^ mice (B6.Cg-Tg(Tagln-cre)1Her/J) were purchased from Jackson Laboratory [25,64]. Animals were maintained on a C57Bl/6 background, kept in ventilated cages with a 12 h day/night cycle, and fed standard mouse chow and water. Genotyping of the animals was performed by PCR (primers used for genotyping are depicted in Supplementary Table S6). Conditional mutant mice with the genotype SM22a^cre/+^ x CXCL12^flox/flox^ displayed reduced survival with 50% of mice dying perinatally; the surviving mice reached adulthood. Age-matched CXCL12^flox/flox^ littermate mice were used as controls. For rescue experiments, 3-week-old mutant mice and control mice were treated with the CXCR7 agonist TC14012 (10 mg/kg) intraperitoneally once in every 6 days for 5 weeks. This dosage regimen and time course was selected based on previous studies in mice [6,61]. Animals were checked daily for signs of stress and pain throughout the experiments. If such signs as shaggy fur or emaciation were observed, the animals were additionally treated with dipidolor (10 mg/kg, s.c.). After conduction of the experiments, mice were euthanized by cervical dislocation and organs were harvested for qRT experiments and Western blot.

### 4.2. High-Resolution Episcopic Microscopy (HREM)

SM-CXCL12^−/−^ mouse embryos were harvested at embryonic day E 14.5 and 17.5, fixed in 4% PFA/PBS for at least 1–2 days, and processed for HREM data generation according to standardized protocols [65,66,67]. In short, samples were washed in PBS for one day, dehydrated in a series of ethanol with increasing concentrations (30%, 50%, and 70% for 24 h; 80% for 16 h; 90% for 3–4 h; and 100% for 4–6 h, two changes), infiltrated with Solution A containing 1.25 g benzoyl peroxide, plasticized (Catalyst) with a methacrylate resin kit (JB-4 embedding kit, Polysciences Europe GmbH) and 0.4 g eosin per 100 mL (3 days, two changes), and finally embedded in this solution after addition of Solution B as previously described [65,68]. After polymerization for 2 days under anaerobic conditions at room temperature, blocks were baked at 80 °C for one day and subjected to HREM data generation using an OHREM apparatus (Indigo Scientific, Baldock, UK) following a standard protocol [66,67]. Resulting HREM data consisted of stacked series of 2000–4000 single digital images with an isotropic voxel size of 3 µm. 3D volume models were produced immediately from the HREM volume; 3D surface models were produced after manual segmentation. Amira 6.7 (Thermo Fisher Scientific, Waltham, MA, USA) was used for data processing, visualization, and analysis.

### 4.3. Echocardiography

Echocardiography was performed in 20-week-old age-matched mutants and controls using a Vevo 2100 Imaging System (VisualSonics Inc., Toronto, Canada) with a 30-MHz high-frequency ultrasound transducer. For the rescue experiments with the CXCR7 agonist TC14012, echocardiography was performed in 8–10 weeks old controls (*n* = 10), cKO mice with sham treatment (*n* = 10), and cKO mice treated with TC14012 (*n* = 10). Mice were anaesthetized with continuous isoflurane flow (0.5% and 99.5% O^2^) over a face mask and were fixed on a temperature-controlled (37.5 °C warm) pad. Ejection fraction (EF) was calculated with the LV-tracing method measuring left ventricular intercavitary areas at end-diastole and end-systole in the parasternal long-axis view (PLAX). M-Mode recordings measuring end-systolic (LVESD), end-diastolic LV diameters (LVEDD), intraventricular septum thickness (IVSD), and LV posterior wall thickness (LVPWD, LVPWS) were obtained in PLAX at the papillary muscle level. The sonographers were blinded to the genotypes. Mice received additional analgesia with dipidolor (piritramid, 10 mg/kg KG) if needed. After conduction of the experiments, mice were euthanized by cervical dislocation.

### 4.4. Quantitative RT-PCR in Heart Tissue and Human Cells

Human microvascular endothelial cells (HMEC-1; ATCC^®^ CRL-3243™) and human aortic vascular smooth muscle cells T/G (T/G HA-VSMC (ATCC^®^ CRL-1999™)) were cultured according to the manufacturer protocol. Mouse heart tissue and HMEC-1 and HAoSMC cells were harvested, washed in 1× PBS, and homogenized in TRIzol reagent (Invitrogen, USA), and total RNA was isolated according to the manufacturer’s instructions. Total RNA was reverse-transcribed to cDNA using the QuantiTect RT kit (Qiagen). Exon spanning primers for murine CXCL12, CXCR4, CXCR7, ANP, BNP, COL8A1, CXCL14, RETNLA, VCAN, BGN, BACT, and GAPDH were designed as listed in Supplementary Table S7. Using 2× SYBR green master mix (Applied Biosystems, Foster City, CA, USA), quantitative gene expression was calculated using the comparative ΔΔCt− method with β-actin and GAPDH as a reference gene.

### 4.5. Western Blot

For total protein extraction, heart tissue was homogenized in RIPA lysis buffer, and protein concentration of samples was measured using the BCA protein assay kit (Pierce). In total, 50 µg of protein samples was denatured in SDS loading buffer (Roth, Karlsruhe, Germany) by incubation at 95 °C for 5 min. Protein lysates were separated on SDS-PAGE and blotted to PVDF membranes (Amersham). The membranes were incubated in blocking solution (5% BSA in Tris-buffered saline with 0.1% Tween-20) for 1 h prior to overnight incubation of the membranes with the following primary antibodies at 4 °C: CXCL12 antibody (CST—3740S), CXCR4 (Abcam—ab124824), CXCR7 (Sigma—SAB4502446), AKT (CST—4691S), Phospo-AKT (CST—4060S), ERK (CST—4695S), Phospho-ERK (CST—4370S), RhoA (CST—2117S), Phospho-RhoA (Abcam—ab41435), and GAPDH (Abcam—ab8245). The membranes were then incubated with an appropriate HRP-conjugated secondary antibody (Pierce, Waltham, MA, USA). Blots were visualized utilizing ECL™ Prime Detection Reagent (GE Healthcare, Little Chalfont, UK) and ChemicDoc MP Imaging system (Biorad; Hercules, CA, USA).

### 4.6. CXCL12 ELISA

Plasma was prepared and CXCL12 levels were quantified using a commercially available ELISA kit (R&D systems, Minneapolis, MN, USA, Mouse CXCL12/CXCL12 alpha Quantikine ELISA Kit) following manufacturer’s instructions.

### 4.7. Histology and Immunostaining

Hearts were excised, fixed in 4% formalin overnight at 4 °C, and embedded in paraffin according to standard histological methods. The hearts were sectioned in 3–5 μm thin longitudinal and transversal slices. Paraffin sections were stained with hematoxylin and eosin (H&E) to analyze histopathology. To evaluate collagen deposition, sections were stained with picrosirius red stain kit (Polysciences, Inc, Warrington, PA, USA) and quantified by Image J software. Ferret diameters and cross-sectional areas of cardiomyocytes were analyzed after staining cell membranes with Texas Red™-X conjugated antibodies against wheat germ agglutinin (WGA; Invitrogen, Waltham, MA, USA, 1:100). Twenty-five cardiomyocytes per field and 10 fields for each section were analyzed using Image J analysis software. Capillaries were stained with antibodies against CD31 (Santa Cruz—SC1506, 1:50), and AEC was used as the chromogen. Arteriole density was measured using a SM22a antibody (Abcam—ab14106, 1:200). Apoptotic cells were detected using the TUNEL assay (DeadEnd™ Fluorometric TUNEL System, Promega). Sections were co-stained with DAPI to detect all cell nuclei. For quantification, the apoptotic index (AI) was calculated as the percentage of TUNEL+ nuclei (green) to total nuclei DAPI (blue). Cardiomyocyte proliferation was detected using pH3+ antibody (Merck—06–570, 1:500) and the percentage calculated to total nuclei (DAPI). Digital photographs were taken at a magnification of 400×, and four random high-power fields (HPFs) of each heart sample (*n* = 6) were analyzed utilizing NIH Image J software. For immunofluorescence analyses, hearts were embedded in optimal cutting temperature compound (OCT). Cryosections with 10 μm thickness were prepared and immunostained using standard techniques. Cryosections were stained with the following antibodies: CXCL12 (R&D systems, IC-350G, 1:10), CXCR4 (Santacruz, Santa Cruz, CA, USA, SC-53534-AF488, 1:50), CXCR7 (Novus Biologicals, NBP2-24779AF488, 1:100), PECAM-1 (Santa Cruz, SC1506, 1:50), smooth muscle protein 22-alpha (SM22a) (Abcam, Cambridge, UK, ab14106, 1:200), and Sarcomeric α-Actinin (Sigma, St. Louis, MO, USA, A7811, 1:500). Following immunostaining, sections were embedded into ProLong™ Glass Antifade Mountant with NucBlue™ (Hoechst 33342, Thermofisher Scientific, Waltham, MA, USA). Sections were analyzed under Zeiss fluorescence microscopy and images were acquired with a Zeiss AxioCam (Carl Zeiss Microscopy GmbH, Jena, Germany).

### 4.8. Flow Cytometric Analyses of Spleen, BM and Heart

Three- to four-month-old age-matched control and mutant murine hearts and spleens were isolated, washed in 1× PBS, and placed in fresh ice-cold HBSS. Tissues were digested and single cell suspensions were isolated as previously described [69]. BM cells were collected by gently flushing the tibia and femur with ice-cold 1× HBSS solution and followed the protocol as previously described [51]. Cell suspensions were filtered using a 40 μm cell strainer and centrifuged at 1500 rpm for 5 min at 4 °C. Cell pellets were resuspended in 1× Red Blood Cell Lysis Buffer (Biolegend, San Diego, CA, USA) and washed twice with cell staining buffer solution (Biolegend). Before staining with various antibodies, cells were treated with FC receptor blocking with TruStain FcX™ PLUS (anti-mouse CD16/32 Antibody; Biolegend) for 5 min on ice. The following list of BD biosciences and Biolegend antibodies were used for analysis: CD45-BV510 (Clone 30-F11, 1:25), CD19-BV650 (Clone 6D5, 1:10), CD90-BUV395 (Clone 30-H12, 1:33), F4/80-PE (Clone BM8, 1:10), Gr-1-BV421 (Clone RB6-8C5, 1:100), CD184-FITC (Clone 2B11, 1:10), CD11b-BV785 (Clone M1/70, 1:50), and CD206-APC (Clone C068C2, 1:10). For cell viability, 7-AAD (#420403) was added 5 min before measurement. For fluorescence compensation, all isotypes of the mouse were used with AbC™ Total Antibody Compensation Bead Kit (#A10497). Gates were set with the help of fluorescence minus one control. Samples were measured on a FACSymphony A5 flow cytometer (BD biosciences), and data were analyzed using FlowJo software v9.9.6 (FlowJo, Ashland, OR, USA).

### 4.9. RNA Sequencing

Library preparations and sequencing were performed at the Institute of Genomics and RNomics, Biocenter, Innsbruck Medical University. For RNA sequencing, total RNA from mouse hearts was extracted with Qiagen RNeasy mini kit (Qiagen GmbH, Hilden, Austria), quality validated with the Agilent Bioanalyzer (Agilent Technologies, Waldbronn, Germany), and submitted to library preparation with the Quantseq 3’-mRNA-seq library kit (Lexogen GmbH, Vienna, Austria). The resulting libraries were sequenced with an Ion Proton sequencer using PI chips and Hi-Q chemistry (Thermo Fisher, Vienna, Austria) at a minimum of 25 million quality filtered reads per library. Fastq files were mapped with a *STAR* + *bowtie* pipeline against an mm10 reference build file of 43,280 known murine transcripts. Raw counts were RPM + mean total count normalized and subjected to an “all-zero” and “single count” filter, leaving 22,169 unique transcripts. Before further analysis, the remaining normalized raw counts were regularized logarithm-transformed, providing a roughly homoscedastic distribution. Differential expression was calculated using the *DEseq2* package, filtered by all genes with *p* < 0.05 after Benjamini–Hochberg (BH) multiple testing correction and ascendingly ordered by fold-change. Hierarchical clustering was performed using the *pheatmap* package (euclidean distance). The KEGG (Kyoto Encyclopedia of Genes and Genomes) and Reactome pathway enrichment analyses of the differentially expressed genes were conducted by the Enrichr (https://maayanlab.cloud/Enrichr/enrich?dataset=9ae3742d8fd7ee20209051133e10491c, access date 30 May 2021) online bioinformatics tool. For predicting protein–protein interactions (PPI) and constructing the PPI network, the STRING database (http://string-db.org, access date 30 May 2021) was employed. Sequencing datasets were deposited at the NCBI GEO SRA database with accession number PRJNA648836.

### 4.10. Statistical Analysis

Data were presented as mean ± SD. Data were analyzed statistically using the GraphPad Prism 8 software (Graph Pad Software, La Jolla, CA, USA). Multiple group comparisons were performed by one-way analysis of variance (ANOVA) followed by the Tukey’s and Sidak multiple comparisons test. Comparisons between the two groups were performed using the unpaired two-sided Student’s *t*-test. *p* ≤ 0.05 was considered statistically significant.

## 5. Conclusions

In our study, we show for the first-time evidence implicating an important role of SMC derived CXCL12 for coronary artery development and maturation, progression of cardiac hypertrophy, and tissue homeostasis of M2 CD206 macrophages, advancing the understanding of CXCL12/CXCR4/CXCR7 biology in the cardiovascular system. While cKO of CXCL12 in SMCs lead to decreased expression of its corresponding receptor CXCR7 in endothelial cells, treatment with a CXCR7 agonist attenuated cardiac hypertrophy and restored cardiac function in cKO mice. Since CXCL12 was also highly expressed in human smooth muscle cells, pharmacological stimulation of CXCR7 might be a promising target to limit the progression of excessive hypertrophic myocardial remodeling.

## Data Availability

The authors declare that all data generated during this study are included in the article and supplementary files. All other data and methods that support the findings of this study are available from the corresponding author upon reasonable request. RNA Sequencing datasets were deposited at the NCBI GEO SRA database with accession number PRJNA648836.

## Supplementary Materials

The following are available online at https://www.mdpi.com/article/10.3390/ijms22115908/s1.
