# Supplementary material for: Smooth Muscle Specific Ablation of CXCL12 in Mice Downregulates CXCR7 Associated with Defective Coronary Arteries and Cardiac Hypertrophy
Source: Int J Mol Sci. 2021 May 31;22(11):5908. doi: 10.3390/ijms22115908 (PMC8198701; doi:10.3390/ijms22115908)
Supplement: Supplementary file 1 [file ijms-22-05908-s001.zip › ijms-1206857-supplementary.pdf]

ONLINE SUPPLEMENTARY DATA

Smooth muscle specific ablation of CXCL12 in mice downregulates CXCR7 associated with defective coronary arteries and cardiac hypertrophy

Ghadge SK[1 6*], Messner M[1*], Seiringer H[1], Maurer T[1], Staggl S[1], Zeller T[2], Müller C[2], Boernigen D[2], Weninger WJ[3], Geyer SH[3], Sopper S[4], Krogsdam A[5], Poelzl G[1], Bauer A[1], Zaruba MM[1]

(1) Department of Internal Medicine III, Cardiology and Angiology, Medical University Innsbruck, Innsbruck, Austria
(2) Clinic for Cardiology, University Heart and Vascular Center Hamburg, Medical University Center Hamburg-Eppendorf
(3) Division of Anatomy & MIC, Medical University of Vienna, Vienna, Austria
(4) Department of Internal Medicine V, Hematology and Oncology, Medical University Innsbruck, Innsbruck, Austria
(5) Biocenter, Division of Bioinformatics, Medical University Innsbruck, Innsbruck, Austria
(6) Max F. Perutz Laboratories (MFPL), Department of Medical Biochemistry, Medical University of Vienna, Vienna, Austria.

This supplementary informations file contains:

Supplementary Figures and Figure Legends and Supplementary Tables

Figure S1

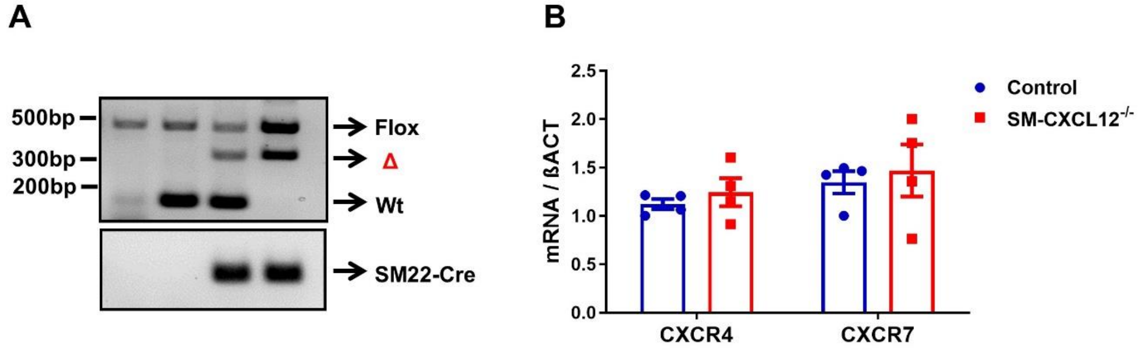

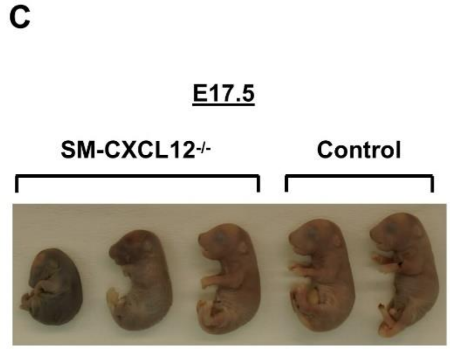

D

Genotype	Observed offspring's	p-value
CXCL12 f/f	42	-
CXCL12 f/f SM22-Cre	12	0.002**

Figure S1: Prenatal mortality with no altered CXCR4 and CXCR7 mRNA expression analysis in the hearts of SM-CXCL12-/- mice. **(A)** PCR genotyping confirming cre specific deletion in cKO mice (Wild type: Wt, floxed: Flox, SDF-1 exon-1 deleted: Δ). **(B)** Bar graph representing the mRNA quantification of the CXCR4 and CXCR7 receptor levels normalized to the β-Actin mRNA expression in hearts of control and SM-CXCL12-/- mice, n=4. **(C)** Macroscopic examination of control and SM-CXCL12-/- E17.5 embryos. **(D)** Table showing an abnormal mendelian ratio of SM-SDF-1-/- offspring's at birth from 6 independent breedings. **$p < 0.01$ from Student's t-test. Data represent mean ± SD.

Figure S2

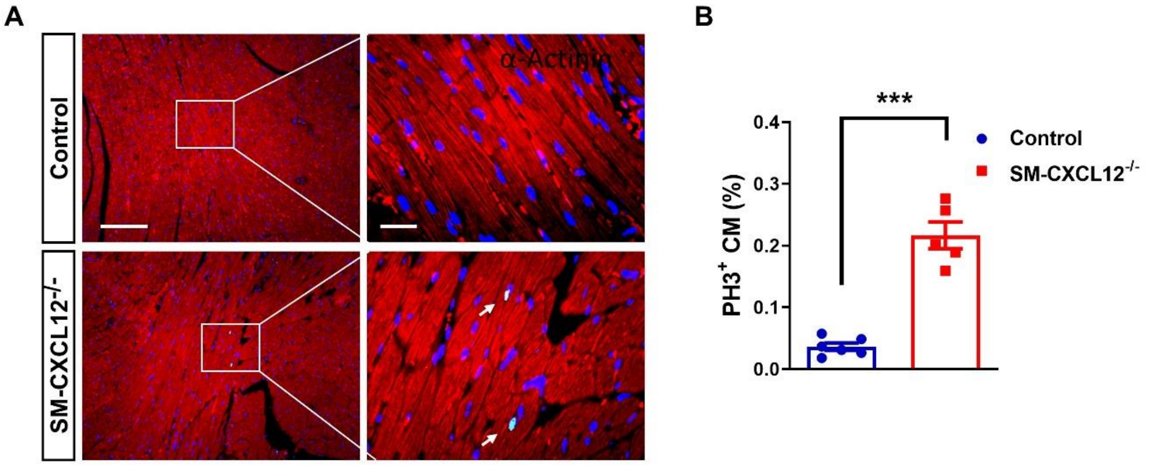

Figure S2: SM-CXCL12-/- mice revealed increased hyperplasia. **(A-B)** Cardiomyocyte DNA replication determined by co-staining of the mitosis marker pH3+ (bright green nuclei marked by arrows) and sarcomeric α-actin (red) of cardiomyocytes in hearts of WT and cKO mice (left panel). Bar graph represents quantification of the percentage of pH3-positive cardiomyocyte nuclei to total cell nuclei (DAPI-blue) (right panel; n=5-6), Scale bars: 200 µm, 20 µm. Data represent mean ± SD; ***p < 0.001 from Student's t-test.

Figure S3

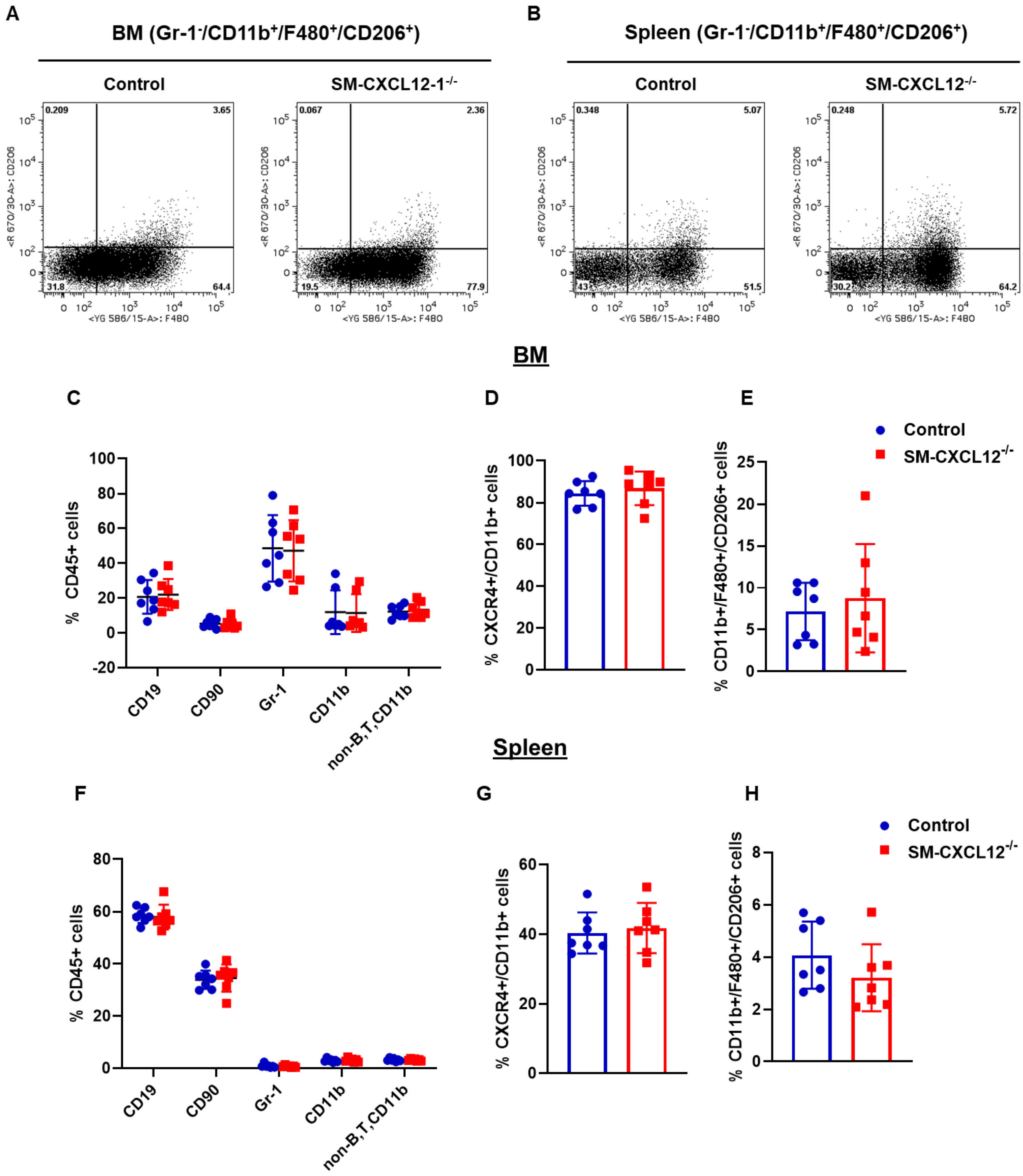

Figure S4: FACS analysis of BM and spleen in SM-CXCL12$^{-/-}$ mice displayed no significant differences in leukocyte cell populations. Representative scatter plots showing M2-like macrophages (Gr-1-/CD11b+/F480+/CD206+) from BM **(A)** and spleen **(B)** of control and SM-CXCL12-/- mice. Bar graphs showing the percentage of CD45+ leukocyte subsets in **(C)** BM and **(F)** spleen for CD19+, CD90+, Gr-1+, and CD11b+ cell populations, n=7. Bar graphs representing the percentage of CXCR4+/CD11b+ cells in the **(D)** BM and **(G)** spleen of control and cKO mice, n=7. Bar graph showing the quantification of Gr-1-/CD11b+/F480+/CD206+ cells in the **(E)** BM and **(H)** spleen of control and SM-CXCL12-/- mice, n=7. Data represent mean ± SD.

Figure S4

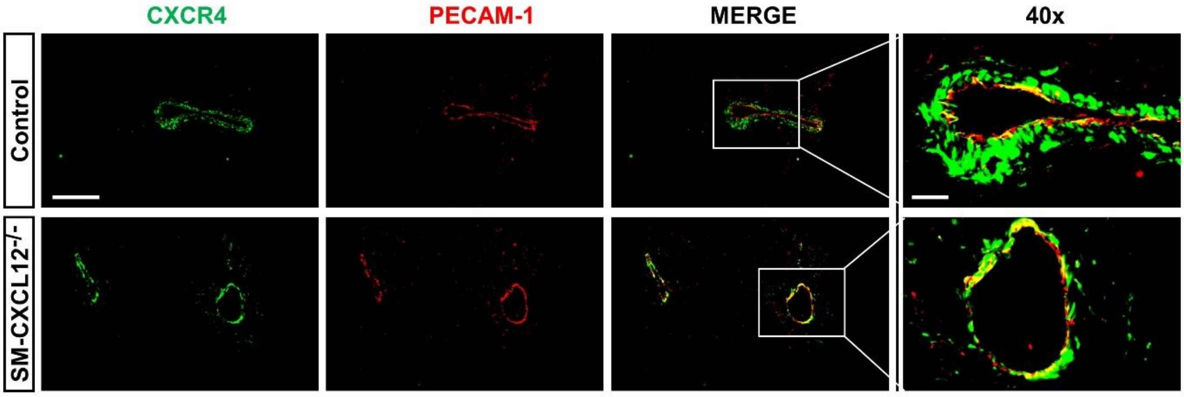

Figure S3: CXCR4 expression is mainly localized in arterial endothelial and the smooth muscle layer. Immunofluorescence staining of CXCR4 co-stained with the endothelial marker PECAM-1 in arteries of control and cKO mice. Conditional deletion of CXCL12 in smooth muscle cells did not affect CXCR4 expression. Scale bar, 200 µm & 20 µm.

Table S1: Mouse Echo measurements

Parameters	WT (n=11)	SM-CXCL12$^{-/-}$ (n=11)	P-value
**Age (weeks)**	21.99 ± 5.53	21.82 ± 5.55	0.983
**EF (%)**	59.36 ± 1.80	40.67 ± 3.58	0.0001
**FS (%)**	17.62 ± 1.00	12.41 ± 1.39	0.008
**SV (µl)**	33.67 ± 2.42	19.75 ± 1.93	0.0002
**IVSD (mm)**	0.85 ± 0.05	1.11 ± 0.07	0.007
**LVPWD (mm)**	0.87 ± 0.03	1.09 ± 0.03	0.0001
**LVEDD (mm)**	3.96 ± 0.11	3.82 ± 0.24	0.590
**LVESD (mm)**	2.66 ± 0.09	2.89 ± 0.27	0.444

Values are Mean ± SD. Ejection fraction (EF), Fractional shortening (FS), Stroke volume (SV), Interventricular septum thickness diastolic (IVSD), Left ventricular posterior wall diameter diastolic (LVPWD), Left ventricular end-diastolic diameter (LVEDD), Left ventricular end-systolic diameter (LVESD)

Table S2: Differentially expressed genes from KO vs. Control hearts

		Fold Change	P-value (BH)
1	Vcp-rs	6,70556937	3,17E-11
2	Vcan	3,86473234	1,88E-07
3	Cnksr1	5,62776172	2,55E-07
4	Dio2	5,0471046	2,55E-07
5	Edn3	7,31541572	2,55E-07
6	Cilp	5,4557627	2,65E-07
7	Col8a1	4,51939324	2,65E-07
8	Cpxm2	3,30967117	3,72E-07
9	Lman1l	6,57661592	4,33E-07
10	Rcan1	4,17861316	4,33E-07
11	2900055J20Rik	6,09992463	1,40E-06
12	Retnla	0,22598469	1,76E-06
13	Nlrc3	5,57473294	4,30E-06
14	Ano10	0,32202441	1,27E-05
15	Svep1	3,12089839	7,48E-05
16	Crlf1	4,91503037	0,00015026
17	Myh7	3,82395585	0,00023362
18	Tceal7	4,57490829	0,00024936
19	Mybpc2	4,05780982	0,00026205
20	1500017E21Rik	2,95493138	0,00051362
21	Ccdc68	3,78637478	0,00076994
22	Gsdma	4,34493427	0,00086992
23	Aqp8	4,12108592	0,00088594
24	Cenpf	0,25321003	0,00098514
25	Aldob	0,2383742	0,00110335
26	Ces5a	3,97174434	0,00128457
27	Atp6v0a4	4,17780246	0,00144353
28	Cxcl14	0,29136409	0,00144353
29	Acaa2	0,41457591	0,00150898
30	Leprel1	3,57517798	0,0016395
31	Adamtsl2	3,07605069	0,00164755
32	Myot	3,91059895	0,00168328
33	Tnfrsf12a	2,97070616	0,00172342
34	Gpr22	0,3585603	0,00182801
35	Slc41a1	0,4237545	0,00182801
36	Dct	2,89961901	0,00241668
37	Cacng6	0,25821211	0,00252339
38	Syt7	0,45005649	0,00275183
39	Ctgf	3,16856845	0,00316839
40	Scml4	2,88284135	0,00316839

41	Gm10364	3,67154219	0,0031994
42	Smim5	0,41320223	0,0031994
43	Thbs4	3,5643406	0,0031994
44	Haus8	2,284575	0,00320777
45	RP24-89F9.4	0,37270802	0,00328087
46	Col1a2	2,06406501	0,00393574
47	Emp1	2,21150066	0,00396968
48	Casq1	3,13165455	0,00399093
49	Xirp2	2,89229235	0,00461658
50	Rasgef1a	3,43797765	0,00481104
51	Pfkfb1	0,36047817	0,00543548
52	Tnip1	2,07024715	0,00572801
53	Fbn1	2,54220181	0,00581095
54	Vcp	0,52278958	0,00597999
55	Adamts8	3,18552244	0,00608045
56	Postn	3,09702754	0,00691237
57	Uck2	2,45191201	0,00701331
58	5830418P13Rik	3,53390495	0,00724471
59	Bcl2	2,75573034	0,00769825
60	Rrp12	2,23745879	0,00769825
61	Bgn	2,27299432	0,00853858
62	Cpne5	0,36588065	0,01018644
63	Gm13889	2,53205978	0,01018644
64	Corin	0,54238269	0,01100193
65	Cbln1	0,29802616	0,01121311
66	Fam198b	2,50461689	0,01125291
67	Fbln2	2,27420801	0,01125291
68	Kif26b	3,0401955	0,01125291
69	Krt18	3,43490458	0,01125291
70	Nrgn	0,31106816	0,01125291
71	RP24-79E2.2	0,43517132	0,01125291
72	Wnk2	0,42428236	0,01129767
73	Esm1	3,16694828	0,011315
74	Plxnb1	0,39322595	0,0123989
75	Vgll2	3,09391585	0,01288285
76	Ahnak2	2,81625172	0,01344984
77	Atg4c	0,31504442	0,01409702
78	Gas2l3	2,9552989	0,01409702
79	Pdlim4	0,42804231	0,01440849
80	Foxo6	0,430327	0,01510744
81	Lmntd1	0,39941585	0,01587873
82	Ppp1r1b	0,34848866	0,01587873
83	Sfrp2	3,0718742	0,01632055
84	Cdh4	2,80690076	0,0176797

85	1500009L16Rik	2,8754482	0,01828082
86	Inhba	3,1882931	0,01828082
87	Itgbl1	2,63769661	0,01828082
88	Rbfox1	0,54377644	0,01828082
89	Prnp	2,60662155	0,01877861
90	Slc36a2	0,50325519	0,0191305
91	Adra1b	0,41361006	0,01918042
92	Syde2	0,37841679	0,02204821
93	Gstt2	0,47684514	0,02213878
94	Opn4	0,39532876	0,02307141
95	Ugp2	2,53129328	0,02307141
96	Nos1	3,16233714	0,02328197
97	Lamc2	2,82380463	0,02450065
98	Clec3b	0,46470107	0,02455492
99	Anxa1	2,90112137	0,02461407
100	Col5a2	2,86487151	0,02461407
101	Pirt	0,39629335	0,02461407
102	Sv2a	0,44666169	0,02461407
103	Gdf15	2,8496739	0,02501845
104	Ptprn	2,68583079	0,02501845
105	Col4a1	1,86397386	0,02568492
106	Pde8a	1,94530863	0,02740975
107	Pdzd3	2,74412696	0,02740975
108	Ankrd45	3,10381486	0,02784643
109	Clic5	1,99880086	0,02828313
110	Ramp1	0,38382804	0,02828313
111	Il15	0,4513116	0,02869324
112	Col4a4	2,05902821	0,02872178
113	D630024D03Rik	3,07582724	0,03018306
114	Acta1	2,88429632	0,0316434
115	Carhsp1	2,03646627	0,0325293
116	Gnao1	2,18643103	0,0325293
117	Etv4	3,00769154	0,03487568
118	Gm6086	0,40188241	0,03487568
119	Impa2	0,4814236	0,03487568
120	Mov10l1	0,50271624	0,03487568
121	Mrgprh	0,37667643	0,03487568
122	Pcbd1	2,81269581	0,03491186
123	Cdv3	2,03160858	0,03527514
124	Npy	0,33197843	0,03580396
125	Gm15543	0,47475008	0,03588354
126	Irx1	0,40615828	0,03588354
127	Itga2b	0,35835802	0,03588354
128	Fgf6	3,00087863	0,03662249

129	Fstl3	2,68138976	0,03737044
130	Iigp1	0,42772027	0,03842604
131	Syndig1	2,85766477	0,04068938
132	Penk	0,3817454	0,04275611
133	Gm12092	2,75251567	0,04317262
134	Emilin2	0,44679781	0,04319327
135	Tprn	2,41767142	0,04319327
136	Hsbp1l1	0,43965223	0,04392996
137	Ankrd2	2,89798754	0,04399058
138	Sorbs2	2,28729552	0,04422599
139	Cd300lb	2,93363295	0,04455722
140	Klhl28	2,62899433	0,04455722
141	Ace	2,29056682	0,04502778
142	Mlf1	0,50127453	0,04502778
143	Col26a1	2,70339307	0,0475078

Table S3: Top 10 KEGG enriched pathways of differentially expressed genes between control and SM-CXCL12 KO hearts

Index	Name	P-value	Adjusted p-value	Odds Ratio	Combined score
**1**	ECM-receptor interaction	0.000002175	0.0006589	11.80	153.80
**2**	Small cell lung cancer	0.0005201	0.07879	7.60	57.48
**3**	Hypertrophic cardiomyopathy (HCM)	0.003381	0.2049	6.51	37.01
**4**	Focal adhesion	0.0005808	0.05866	4.92	36.66
**5**	Protein digestion and absorption	0.003980	0.2010	6.22	34.35
**6**	Relaxin signaling pathway	0.002522	0.1910	5.34	31.94
**7**	Fructose and mannose metabolism	0.02589	0.6538	7.99	29.20
**8**	AGE-RAGE signaling pathway in diabetic complications	0.005988	0.2268	5.54	28.35
**9**	Amoebiasis	0.007090	0.2387	5.28	26.12
**10**	PI3K-Akt signaling pathway	0.004209	0.1822	3.13	17.15

Table S4: Top 10 Reactome enriched pathways of differentially expressed genes between control and SM-CXCL12 KO hearts

Index	Name	P-value	Adjusted p-value	Odds Ratio	Combined score
1	Defective CHST3 causes SEDCJD_Homo sapiens_R-HSA-3595172	0.001041	0.3186	39.96	274.42
2	Defective CHSY1 causes TPBS_Homo sapiens_R-HSA-3595177	0.001041	0.2655	39.96	274.42
3	Defective CHST14 causes EDS, musculocontractural type_Homo sapiens_R-HSA-3595174	0.001041	0.2276	39.96	274.42
4	Assembly of collagen fibrils and other multimeric structures_Homo sapiens_R-HSA-2022090	0.000002342	0.001791	15.54	201.47
5	Collagen biosynthesis and modifying enzymes_Homo sapiens_R-HSA-1650814	0.000005843	0.002235	13.32	160.51
6	Dermatan sulfate biosynthesis_Homo sapiens_R-HSA-2022923	0.002676	0.4095	25.43	150.62
7	Collagen formation_Homo sapiens_R-HSA-1474290	0.000002555	0.001303	11.52	148.32
8	Signaling by Activin_Homo sapiens_R-HSA-1502540	0.003760	0.4426	21.52	120.13
9	CS/DS degradation_Homo sapiens_R-HSA-2024101	0.004367	0.4772	19.98	108.57
10	Extracellular matrix organization_Homo sapiens_R-HSA-1474244	1.286e-7	0.0001968	6.42	101.94

Table S5: Mouse Echo measurements

Parameters	WT (n=10)	SM-CXCL12$^{-/-}$ (n=10)	SM-CXCL12$^{-/-}$ + TC14012 (n=10)
**Age (weeks)**	8.81 ± 0.21	8.94 ± 0.24	9.06 ± 0.53
**EF (%)**	55.99 ± 4.10	40.97 ± 9.67$^{###}$	52.24 ± 8.46**
**FS (%)**	16.47 ± 4,29	12.54 ± 5.59	15.45 ± 3.90
**SV (µl)**	25.07 ± 10.4	24.00 ± 10.31	25.84 ± 9.17
**IVSD (mm)**	0.79 ± 0.10	1.15 ± 0.18$^{###}$	0.96 ± 0.15§*
**LVPWD (mm)**	0.82 ± 0.08	1.07 ± 0.21$^{##}$	0.87 ± 0.12*
**LVEDD (mm)**	3.54 ± 0.44	4.21 ± 1.18	3.85 ± 0.65
**LVESD (mm)**	2.45 ± 0.38	3.24 ± 1.33	2.78 ± 0.63

Values are Mean ± SD.
*P≤0.05 SM-CXCL12 KO vs. SM-CXCL12KO + TC14012
**P≤0.01 SM-CXCL12 KO vs. SM-CXCL12KO + TC14012
##P≤0.01 Control vs. SM-CXCL12KO
###P≤0.001 Control vs. SM-CXCL12KO
§P≤0.05 Control vs. SM-CXCL12KO + TC14012
Ejection fraction (EF), Fractional shortening (FS), Stroke volume (SV), Interventricular septum thickness diastolic (IVSD), Left ventricular posterior wall thickness diastolic (LVPWD), Left ventricular end diastolic diameter (LVEDD), Left ventricular end systolic diameter (LVESD).

Table S6: Primer sequences for genotyping

Gene	Forward primer	Reverse primer
**CXCL12 F4**	5´ATCTCTGGGAGACCTGTTTGG 3´	
**CXCL12 R4**		5´GGACTGCTAGGCTTAGGGCAA 3´
**CXCL12 R5**		5´CAGCGCGAGTTCAAGAGCT 3´
**CXCL12 R7**		5´CGCTATGACGGCAATAAAAAG 3´
**CRE**	5´GCGGTCTGGCAGTAAAAACTATC 3´	5´GTGAAACAGCATTGCTGTCACTT 3´

Expected PCR amplicons: Wt = 180 bp, Deleted = 320bp, Floxed = 480bp

Table S7: Primer sequences for real-time PCR

Gene	Forward primer	Reverse primer
**CXCL12**	5´CATCAGTGACGGTAAACCAG 3´	5´GCACAGTTTGGAGTGTTGAG 3´
**ACTB**	5´CGCCACCAGTTCGCCATGGA 3´	5´TACAGCCCGGGGAGCATCGT 3´
**GAPDH**	5´CGTCCCGTAGACAAAATG 3´	5´TCAATGAAGGGGTCGTTGAT 3´
**BNP**	5´CCAGAGCAATTCAAGATGCAG 3´	5´GGTCTTCCTACAACAACTTCAG 3´
**ANP**	5´TCAACCCACCTCTGAGAGAC 3´	5´ATCGACTGCCTTTTCCTCC 3´
**CXCR4**	5` CGGCTGTAGAGCGAGTGTTG 3`	5` GCAGGGTTCCTTGTTGGAGT 3`
**CXCR7**	5´CACCGTCAGGAAGGCAAACC 3´	5´CAATGCAGTCGCTGCTGTTAC 3´
**COL8A1**	5´CTCTTGGTCCAGGTTCTCCA 3´	5´AAGGAAATCCCACCTGTGC 3´
**CXCL14**	5´CCAAAGTACCCACACTGCGA 3´	5´CTTCGTAGACCCTGCGCTTC 3´
**RETNLA**	5´ACTATGAACAGATGGGCCTCC 3´	5´AACGAGTAAGCACAGGCAGT 3´
**VCAN**	5´CCTGCAATTACCACCTCACCT 3´	5´TGCCCATCTCCCGTTTCCTA 3´
**BGN**	5´CCTGAGTTTTCTGCCTACCCT 3´	5´CCGAAGCCCATAGGACAGAA 3´
